# Simple Rules Reproduce a Hub-Shaped Cell Metabolic Network

**DOI:** 10.1371/journal.pbio.0030261

**Published:** 2005-06-28

A map of a cell's metabolic pathways looks like an airline route map on steroids, with hundreds of reactions forming a complex and interconnected network. And just as a few cities serve as destination hubs for many different flights, a few metabolites, such as ATP and NADH, form biochemical hubs within the metabolic network. These molecules are involved in far more reactions than the average, and thereby serve to couple otherwise unrelated reactions within the cell. How did this hub-shaped network arise? In this issue, Thomas Pfeiffer and colleagues employ a computer simulation of a simplified metabolic system to show that two key features in the evolution of a hub system are enzyme specialization and the transfer of chemical groups between metabolites.

The authors created “molecules” from all possible combinations of seven “groups.” They began the simulation with seven “enzymes” that catalyze the transfer of one group from one molecule to another. Initially, each enzyme was a generalist—it could take a group from any molecule and donate it to any other. This mirrors one plausible scheme for the actual evolution of cellular biochemistry. Over the course of the simulation, enzymes could mutate to preferentially increase their affinity for one substrate at the expense of others. The number of enzyme types could be increased by “gene duplications.” Other parameters allowed the simulated cell to take up and excrete metabolites, and to grow.

As the system evolved, enzymes proliferated and became more specialized, until the final mix included about two dozen enzymes, each of which catalyzed only one or two reactions. As a consequence, some metabolites fell out of use, and the final number of metabolites dropped from 128 to 33. While most took part in only two or three reactions, a few emerged as hubs, participating in eight or more separate reactions. While this mathematical distribution did not match that found in the metabolic network of a whole real cell, it did approximate that of similar-sized sub-cellular metabolic networks. The central importance of group transfer to this structure was brought out when the authors reran the simulation without group transfer. When reactions simply added or removed a group, without transferring it to another molecule, a much simpler network without hubs evolved instead. [Fig pbio-0030261-g001]

**Figure pbio-0030261-g001:**
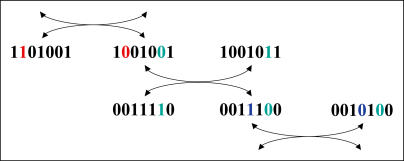
For modeling purposes, metabolites can be denoted as binary strings of biochemical groups; enzymes catalyze the transfer of the groups

This is not the last word on biochemical evolution, but it does show how an initially generalist metabolism can evolve into the highly specialist system found in all existing cells. Further experiments that model known reactions more closely may be useful in elucidating more details of the evolutionary process that led to the emergence of the biochemical “hubbub” that characterizes life today.

